# Delinquency in incarcerated male adolescents is associated with single parenthood, exposure to more violence at home and in the community, and poorer self-image

**DOI:** 10.3325/cmj.2013.54.460

**Published:** 2013-10

**Authors:** Stanislava Erdelja, Petra Vokal, Marija Bolfan, Sergej Augustin Erdelja, Branka Begovac, Ivan Begovac

**Affiliations:** 1 Center for social welfare, Donja Stubica, Croatia; 2 Department of Psychological Medicine, University of Zagreb School of Medicine, University Clinical Hospital, Zagreb, Croatia; 3 Center for social welfare, Krapina, Croatia

## Abstract

**Aim:**

To assess the relationships between delinquency and demographic and family variables, academic performance, war stressors, home/community, school, and media violence exposure, self-image, and psychopathology.

**Methods:**

This cross-sectional study included 100 delinquent, incarcerated male adolescents and 100 matched schoolchildren from Croatia. It lasted from January 2008 to June 2009, and used socio-demographic questionnaire, questionnaire on children’s stressful and traumatic war experiences, exposure to violence scale, the Offer Self-Image Questionnaire, and Youth Self-Report Questionnaire.

**Results:**

Logistic regression analysis showed that delinquency in incarcerated adolescents was more likely related to having parents who did not live together (odds ratio [OR] 2.40; confidence interval [CI] 1.18-4.90, *P* = 0.015), being more exposed to violence at home/community (OR 3.84; CI 1.58-9.34, *P* = 0.003), and having poorer self-image (OR 1.09; CI = 1.03-1.16, *P* < 0.002).

**Conclusion:**

Preventive and therapeutic interventions in incarcerated delinquents should be specifically targeted toward single parenthood, family factors, trauma oriented interventions, and focused on multiple dimensions of self-concept of adolescents.

Delinquency is associated with many risk factors, including demographic, genetic, and family characteristics (single parenthood) or academic performance. Many studies have focused on exposure to various forms of violence – in the family or home; community and neighborhood; in school and peer groups; and the media, but other risk factors have also found to be important, such as poorer self-image, various forms of psychopathology, and social characteristics (neighborhoods characterized by poverty) (1-15). Most of the studies dealing with delinquency aim to develop therapeutic interventions in relation to the obtained factors or mediators (9,11,16).

There are relatively few studies on incarcerated adolescents. Many report on delinquents’ traumatic experiences, posttraumatic stress disorder, and importance of developmental tasks of adolescence and parental monitoring (14,17,18). Therapeutic interventions are specifically directed toward assessment and intervention of trauma and psychopathology, and family interventions are used very often.

There are not many studies on delinquents in Croatia and most of them deal with a model that takes into account the interplay between protective and risk factors (19-21). Factors that are often mentioned are parental distrust and punishment, and family dysfunctionality (22-24). The prevalence of delinquency in the last few years has not been reducing (25), which suggests that the current preventive and therapeutic efforts have not been sufficient (25). Another important factor that has to be considered when studying delinquency in Croatia is the influence of Croatian War for Independence 1991-1995. The relationship between war experiences (direct or indirect) and the development of delinquency in adolescents has been relatively rarely described, with contradictory findings. Some studies found no association between the impact of war and bullying (26), whereas others found a relationship between aggressiveness in child refugees and their past war experiences (27) or experiences of their parents, war veterans (28). Besides war-related violence, we expected that delinquency was related to the exposure to other types of violence, eg, violence at home (29). Finally, we also expected an association with poorer self-image (8) and the presence of significant psychopathological syndromes (7).

Our aim was therefore to examine the relationship between demographic, family factors, academic performance, exposure to violence in different contexts (home, community, school, media, war related stress), psychopathology, and delinquency.

## Methods

### Participants

This study was conducted from January 2008 to June 2009 and included delinquent male adolescents aged 14-18 years. We made a preliminary calculation of study power with 80 participants (8) using the methodology of Offer et al (30,31) and obtained excellent results; for example, for the Physical Self-image scale, the resulting value was 0.94. Therefore, it was determined that the study needs to include a minimum of 80 participants.

The participants were incarcerated in two correctional institutions, Pahinsko-Ivanec (N = 55) and Dugave-Zagreb (N = 52), both in Croatia. The adolescents had been placed in these institutions for at least 3 months but not longer than 2 years, and their mean IQ was average for Croatian adolescents in general (21). We assessed about 70% of all delinquents placed in these institutions in two school years. Both institutions were considered as one sample, because their profile of intervention is very similar and they have similar reasons for referral from the court. These two institutions were usually considered similar in previous studies as well (21). The participation rate was 96.15%. A total of 107 adolescent delinquents were contacted. The exclusion criterion was illiteracy, on the basis of which three adolescents were excluded. Four adolescents refused to participate, so the final sample consisted of 100 participants.

The control group was a convenient sample consisting of 100 adolescents, matched on the basis of age, sex, education of parents, and their own education. They were students of Oroslavje high-school who attended vocational programs (carpenter, car mechanic, car body painter, or metal trades). School authorities together with the first author chose adolescents from six classes according to matching criteria. The exclusion criterion was incarceration in a correctional institution, because of which no one was excluded. None of the control participants refused to participate.

### Procedure

The purpose of this study was explained to the parents and adolescent and written informed consent was obtained from parents or caretakers. This investigation lasted two school hours. Instruments were based on self-rating and the data were acquired using a group method of data collection.

The study complied with ethical standards, Croatian laws, and international conventions. It was approved by the Ethics Committee, University of Zagreb, the Ministry of Health and Social Care (Administration of Social Welfare), and the Ministry of Education of the Republic of Croatia.

### Measures

*Socio-demographic data. G*eneral questionnaire about demographic and family data and academic performance was designed for the purposes of the study. Academic performance was generally divided into 5 categories (5 is the best).

*War stressors*. Stressors were assessed using the questionnaire on children’s stressful and traumatic war experiences, a self-administered questionnaire developed and used in Croatia in 1994 with good psychometric properties (32); it was also used later in a modified form, which has also been shown to have good psychometric properties (33). The instrument included the most frequent war stressors and subscales of stressful events: general war events, loss of home, refugee status, victimization of family members, witnessing victimization, and personal victimization. This instrument contains 20 “yes” or “no” questions. The total score is the sum of “yes” responses, representing the intensity of stressful events and ranging from 0 to 20. The Cronbach alpha coefficient of this scale for this study was 0.837.

*Exposure to violence*. A self-rated questionnaire was used to assess the exposure to violence (34). The questionnaire was translated to Croatian using the process of translation-back-translation, with the permission of the author. The questionnaire has been used in international studies and showed good psychometric properties (34). It is an 81-item self-report questionnaire; 6 questions are about demographic data, while other questions deal with the degree of exposure to violence and screen for adolescents who experienced negative emotional consequences and/or sought professional help. The questions are designed to assess the degree of exposure and related psychological reactions in three areas (media, home/community, and school) and are rated on a scale ranging from 1 (not at all) to 5 (a lot). Only subscale totals were cited in this study, according to the conducted factor analysis; so the specific questions were not used in further analysis.

Similar to the original questionnaire, subscales were divided into three areas: media, home/community, and school (34). Additionally, in the factor analysis the media scale was divided into three subscales and home/community scale into two subscales, which made a total of six subscales: “Exposure to violence in media” (12 items); “Symptoms after exposure to violence in media” (9 items); “Seeking help after exposure to violence in media” (3 items); “Exposure to violence in home/community” (22 items); “Symptoms and seeking help after exposure to violence in the home/community” (12 items); “Exposure to violence in school” (16 items). The overall scores for the scales ranged from 1 to 5. The Cronbach alpha for these subscales ranged from 0.861 to 0.955.

*Self-image*. The Croatian version of The Offer Self-Image Questionnaire (a self-administered test comprising 130 items) was used (30,31). This questionnaire has already been used in Croatia and showed good psychometric properties (33). The items were rated on a 6-point Likert scale, with 1 representing “this is a very accurate description of me” and 6 representing “this does not describe me at all.” The whole questionnaire consists of six scales: “Psychological self” (the total scores range from 9 to 60), “Social self” (the total scores range from 9 to 60), “ Sexual attitudes” (the total scores range from 10 to 60), “Family relations” (the total scores range from 19 to 114), “Adaptable self” (the total scores range from 10 to 84), and “Total Offer score” (the total scores range from 9 to 114). The Cronbach alpha coefficients for the scales employed in this study ranged from 0.583 to 0.847.

*Psychopathology*. We used the Croatian version of the Youth Self-Report (YSR) questionnaire (35), which has been used in previous studies in Croatia and has been shown to have good psychometric properties (36). The questionnaire has two parts. The first part assesses the competencies and the second assesses psychopathology. The second part contains items describing behavior, which are rated on a 3-point scale: 0 = not true, 1 = somewhat or sometimes true, 2 = very true or often true. We used the second part of because of its larger clinical relevance, and since it has been used in similar international studies (37). These ratings were combined to form eight narrow band scales or syndromes, two broadband scales, and a “Total Problems” score. The eight syndromes are withdrawn/depressed; somatic complaints; anxious/depressed; social problems; thought problems; attention problems; rule-breaking behavior; and aggressive behavior. Broadband scales are termed “Internalizing” and “Externalizing.” The “Internalizing” scale reflects internal stress and consists of “Withdrawn/Depressed,” “Somatic Complaints,” and “Anxious/Depressed” scales. The “Externalizing” scale reflects one’s conflict with other people and their expectations and consists of “Rule-Breaking Behavior” and “Delinquent Behavior” scales. The Cronbach alpha coefficients of the syndrome scales employed in this study ranged from 0.768 to 0.913.

### Statistical analysis

Data are expressed as frequencies for categorical variables and as means and standard deviations for quantitative variables. The Mann-Whitney test was used to compare quantitative variables, and Fisher exact test or χ^2^-tests were used to compare categorical variables. Associations between continuous variables were assessed using Pearson correlation coefficients. Cronbach’s alpha was used to examine the internal consistency of the psychometric test scores. Factor analysis was made for the “Exposure to violence” scale.

Multivariate analysis was employed to compare delinquent adolescents and controls. All variables that were significant in the univariate analysis (*P* < 0.05) were included in the model. Potential confounding variables were screened using bivariate analysis, including those with *P* < 0.05 in initial models. Considering the high correlations of “Internalizing” and “Externalizing” scales with the “Total YSR problems” scale (r = 0.89 and 0.93, respectively), this scale was included in the model. Considering the high correlations between the Offer subscales and the scale of “Total Offer score” (the scale of “Sexual attitudes” had a Pearson coefficient of 0.42, while the other 4 subscales had Pearson coefficients from 0.83 to 0.92), only the “Total Offer score” scale was included.

Participants were categorized into two age-groups according to the 50th percentile: younger (14-16 years) and older (17-19 years). Participants were also categorized in terms of the academic performance: poorer performance (grades 1-3) and better performance (grades 4-5). Results on “Exposure to violence at home/community” and “Symptoms and seeking help after the exposure to violence at home/community” were also categorized into higher and lower values. The variable of “Total YSR problems” was categorized into 3 categories: highest values, medium values, and lowest values. The adequacy of the final models was assessed using Hosmer-Lemeshow test and checked for linearity, multicollinearity, and outliers. All analyses were performed using SAS version 9.1.3 (SAS institute, Inc., Cary, NC, USA); the level of significance was set at *P* < 0.05.

## Results

### Socio-demographic characteristics

Mean age of delinquent adolescents was 16.76 ± 1.28 years and of controls 16.38 ± 1.22 years. The father’s educational level was used as the measure of socioeconomic status, although it is not quite a precise measure. Approximately 28% of the fathers finished only primary school (up to 8 years of education), approximately 53% finished high school (up to 12 years of education), and approximately 18% had college, university, or a higher degree (more than 12 years of education).

### Comparison of general data, family characteristics, and academic performance

Delinquent adolescents were significantly older than controls (*P* = 0.028, Mann-Whitney test). Significantly more delinquent adolescents than controls had parents who were not living together (*P* ≤ 0.001, Fisher exact test). They also had significantly poorer academic performance (2.97 ± 0.84 vs 3.21 ± 0.94, *P* = 0.013, Mann-Whitney test). Other variables showed no significant relationships (Table 1).

**Table 1:** Socio-demographic data, family characteristics, and academic performance of deliquent adolescents and controls

	Delinquent adolescents (N = 100)	Controls (N = 100)	*P*
Age in years (mean ± standard deviation)	16.76 ± 1.28	16.38 ± 1.22	0.028*
Parents' relationship (No.)			
live together	48	76	≤0.001^†^
do not live together	47	22	
unknown	5	2	
Education of father (No.)			
up to 8 y	33	24	0.249^‡^
up to 12 y of schooling	52	54	
more than 12 y	15	22	
Number of biological siblings (median, range)	1.50 (0.00-10.00)	1.00 (0.00-6.00)	0.124*
Development problems in childhood (No.)			
no	74	77	0.875^‡^
yes	13	12	
unknown	13	11	
Medical disease of adolescents (No.)			
no	69	82	0.088^‡^
yes	23	12	
unknown	8	6	
Academic performance (mean ± standard deviation)	2.97 ± 0.84	3.21 ± 0.94	0.013*

*Mann- Whitney test.

†Fisher exact test.

^‡^χ^2^-test.

### Comparison of war stressors and multi-contextual violence exposure

Compared to controls, delinquent adolescents had significantly greater exposure to violence at home and in the community (*P* = 0.001, Mann-Whitney test), exhibited significantly more violence-related symptoms and help-seeking behavior (*P* = 0.018, Mann-Whitney test), and had significantly greater exposure to violence in school (*P* = 0.004, Mann-Whitney test). Other variables showed no significant relationships (Table 2).

**Table 2:** Comparison of exposure to war, trauma and violence

	Delinquent adolescents (N = 100)	Controls (N = 100)	P*
War stressors (median, range)	3.00 (0.00-14.00)	2.00 (0.00-14.00)	0.878
Exposure to violence in media (mean ± standard deviation)	3.83 ± 0.89	3.77 ± 0.76	0.392
Symptoms after exposure to violence in media (median, range)	1.11 (1.00-5.00)	1.22 (1.00-4.11)	0.521
Seeking help after exposure to violence in media (median, range)	1.00 (1.00-5.00)	1.17 (1.00-5.00)	0.558
Exposure to violence in the home/community (mean ± standard deviation)	1.80 ± 0.75	1.46 ± 0.71	0.001
Symptoms and seeking help after exposure to violence in home/community (mean ± standard deviation)	1.48 ± 0.65	1.30 ± 0.59	0.018
Exposure to violence in school (mean ± standard deviation)	2.01 ± 0.72	1.77 ± 0.72	0.004

*Mann- Whitney test.

### Comparison of self-image and psychopathology

Delinquent adolescents scored poorer than controls on most subscales of self-image, except on the “Sexual attitudes” subscale. On all YSR scales, delinquent adolescents had higher values than controls (Table 3).

**Table 3:** Comparison of self-image and psychopathology

	Delinquent adolescents (N = 100)	Controls (N = 100)	P*
Psychological self (mean ± standard deviation)	28.93 ± 6.81	24.57 ± 5.78	≤0.001
Social self (mean ± standard deviation)	30.10 ± 5.76	26.92 ± 5.58	≤0.001
Sexual attitudes (mean ± standard deviation)	27.03 ± 7.67	25.85 ± 6.78	0.250
Family relations (mean ± standard deviation)	65.88 ± 14.66	54.87 ± 15.11	≤0.001
Adaptable self (mean ± standard deviation)	39.75 ± 6.99	36.36 ± 6.01	≤0.001
Total Offer Score (mean ± standard deviation)	35.38 ± 6.29	31.29 ± 5.65	≤0.001
Withdrawn/Depressed (median, range)	8.00 (0.00-23.00)	4.00 (0.00-17.00)	≤0.001
Somatic Complaints (median, range)	6.00 (0.00- 15.00)	4.00 (0.00-10.00)	≤0.001
Anxious/Depressed (median, range)	4.00 (0.00- 17.00)	1.00 (0.00-19.00)	≤0.001
Social Problems (median, range)	6.50 (0.00- 22.00)	3.00 (0.00-14.00)	≤0.001
Thought Problems (median, range)	5.00 (0.00-19.00)	4.00 (0.00-17.00)	0.033
Attention problems (median, range)	8.00 (0.00-17.00)	6.00 (0.00- 14.00)	0.001
Rule-Breaking Behavior (median, range)	13.50 (0.00- 29.00)	7.00 (0.00- 23.00)	≤0.001
Aggressive Behavior (median, range)	14.00 (0.00- 33.00)	9.00 (0.00- 29.00)	≤0.001
Internalizing (median, range)	17.50 (0.00- 52.00)	8.00 (0.00- 42.00)	≤0.001
Externalizing (median, range)	27.00 (0.00-61.00)	16.00 (0.00- 49.00)	≤0.001
Total Youth Self Report Problems (median, range)	65.00 (0.00-177.00)	41.00 (1.00-125.00)	≤0.001

*Mann- Whitney test.

### Predicting delinquency

Multivariate logistic regression analysis showed that delinquency in incarcerated adolescents was more likely related to having parents who did not live together, being more exposed to violence in the home/community, and having poorer self-image (Table 4).

**Table 4:** Comparison of delinquent adolescents and controls using multivariate logistic regression analysis (N = 193)

	Odds Ratio	95% confidence interval	P*
Age			
younger (14-16 y)	1.00		
older (17-19 y)	1.24	0.63- 2.45	0.519
Parents' relationship			
live together	1.00		
do not live together	2.40	1.18- 4.90	0.015
Academic performance			
better	1.00		
worse	2.27	0.98- 5.26	0.054
Exposure to violence in home/community			
lower	1.00		
higher	3.84	1.58- 9.34	0.003
Symptoms and seeking help after exposure to violence in home/community			
lower	1.00		
higher	1.52	0.69- 3.33	0.291
Exposure to violence in school (per one point increase)	1.76	0.95- 3.28	0.071
Total Offer score (per one point increase)	1.09	1.03- 1.16	0.002
Total Youth Self Report Problems score			
lowest	1.00		
highest	2.58	0.92- 7.19	0.070
Total Youth Self Report Problems score			
medium	1.00		
highest	2.77	0.98- 7.81	0.054

*Hosmer- Lemeshov test was 0.589.

## Discussion

We found the following predictors of delinquency: single parenthood, high exposure to violence at home/community, and poorer self-concept, which is in agreement with other studies (1,8,38,39). It is interesting that, contrary to our hypotheses and previous findings (5-7), there was no correlation between delinquency and poor school achievement, trauma exposure in other contexts, and manifest psychopathology.

Our study indicates the importance of single parenthood as a risk factor, which has also been found in other studies (1). Single parents seem to face more difficulties in raising a child/adolescent (38). An interesting finding of our study is the relationship between delinquency and exposure to violence at home/community, while in other contexts, similar findings were not observed. This result confirms that the feeling of basic security at home and in the community is very important for adolescents, which has also been found in other studies (2,29). The loss of such “safe harbor” seems especially threatening and can be related to the development of delinquency. Finally, we found a significant relationship between delinquency and poor self-concept, which is in agreement with other studies (8,39).

We did not observe an association between war as a stressor and delinquency, although some studies in the area (27) have shown long-term consequences of war. Furthermore, the mean value for war stressors obtained in our study (3.5), when compared with that in similar studies conducted in Croatia several years ago (6.16 and 7.81) (32,33) confirms the decrease in the effect of war on adolescents.

In our study, the exposure to violence in school also did not show an association with delinquency, a result contradicting those of other studies (6). The school environment as a variable seems “sufficiently neutral” (38) or can possibly have some protective value. This finding is in agreement with those of other studies, which suggest that the problem of aggressiveness in Croatian schools is moderately pronounced (40). Although there has been a recent trend of relating media to aggressiveness (5), we did not confirm such a relationship.

Finally, no relationship between manifest psychopathology and delinquency was found, which suggests more “discrete psychological problems,” which are measured by self-concept scales.

This study has some limitations. We used cross-sectional design, although longitudinal design would be more suitable to measure temporal changes in the measured variables. Beside this, our study relied exclusively on self-report measures. Also, delinquent adolescent groups from two institutions differed greatly – Pahinsko-Ivanec has an integrated school, while Dugave-Zagreb does not have one. This leads to the conclusion that delinquent adolescents in Pahinsko-Ivanec could be less educated. Finally, the findings are limited to a sample of incarcerated delinquent youths from Croatia and need to be replicated in other settings.

We did not find the association of delinquency with any single factor, but rather an interplay of various individual, family, and socio-cultural factors. Although in Croatia there is a number of studies trying to determine the factors involved in the development of delinquency (20,21), there are no specific and clear implications for prevention and therapy. Family interventions are rarely emphasized (22) and the trauma-focused interventions and interventions that involve multiple domains of personality and self-concept have not gained sufficient attention and still remain to be conducted (9,14,17,18,41).
